# Metabolic Adaptations of CD4^+^ T Cells in Inflammatory Disease

**DOI:** 10.3389/fimmu.2018.00540

**Published:** 2018-03-15

**Authors:** Cristina Dumitru, Agnieszka M. Kabat, Kevin J. Maloy

**Affiliations:** ^1^Sir William Dunn School of Pathology, University of Oxford, Oxford, United Kingdom; ^2^Max Planck Institute of Immunobiology and Epigenetics, Freiburg im Breisgau, Germany

**Keywords:** Th cells, inflammation, metabolism, microenvironment, Th1 cells, Th17 cells, Th2 cells, regulatory T cells

## Abstract

A controlled and self-limiting inflammatory reaction generally results in removal of the injurious agent and repair of the damaged tissue. However, in chronic inflammation, immune responses become dysregulated and prolonged, leading to tissue destruction. The role of metabolic reprogramming in orchestrating appropriate immune responses has gained increasing attention in recent years. Proliferation and differentiation of the T cell subsets that are needed to address homeostatic imbalance is accompanied by a series of metabolic adaptations, as T cells traveling from nutrient-rich secondary lymphoid tissues to sites of inflammation experience a dramatic shift in microenvironment conditions. How T cells integrate information about the local environment, such as nutrient availability or oxygen levels, and transfer these signals to functional pathways remains to be fully understood. In this review, we discuss how distinct subsets of CD4^+^ T cells metabolically adapt to the conditions of inflammation and whether these insights may pave the way to new treatments for human inflammatory diseases.

## The Inflammatory Microenvironment

It has long been appreciated that leukocytes need to adapt their metabolism to survive and proliferate in the hostile inflammatory environment. However, in recent years, there has been a growing understanding of the complex relationship between the T cell metabolic machinery and their immune function. Metabolic adaptations of T cells go beyond facilitating survival—they are also critical for T cell differentiation and immune effector function (1–4). The complicated interplay between local environment, T cell metabolism, and immune functions remains incompletely understood. In this review, we discuss how CD4^+^ T cells adapt to conditions of inflammation. We first consider how metabolic conditions in inflammatory microenvironments differ from those present in healthy tissues and lymphoid organs. We then summarize the metabolic pathways involved in T-cell activation, followed by discussion of recent studies examining the role of nutrients, oxygen, and temperature on CD4^+^ T cell differentiation and function during inflammation. We further explore how dysregulation of catabolic processes, such as autophagy, can alter the availability of nutrients and lead to aberrant immune responses. Finally, we look at how understanding the metabolic adaptations of CD4^+^ T cells in response to environmental factors may pave the way to new treatments for human inflammatory diseases.

A controlled and self-limiting acute inflammatory reaction is largely beneficial; however, in chronic inflammation, the response becomes dysregulated and prolonged, leading to excessive tissue destruction (5). Chronic inflammation can also develop as an independent response with entirely different pathogenesis, time-course, and clinical manifestations (6). This persistent type of inflammation is associated with many diseases, including rheumatoid arthritis (RA), asthma, celiac disease, or inflammatory bowel disease (IBD). Moreover, several chronic conditions, including obesity, diabetes, cardiovascular disease, or cancer, are known to have inflammatory components (7).

**Figure 1:**
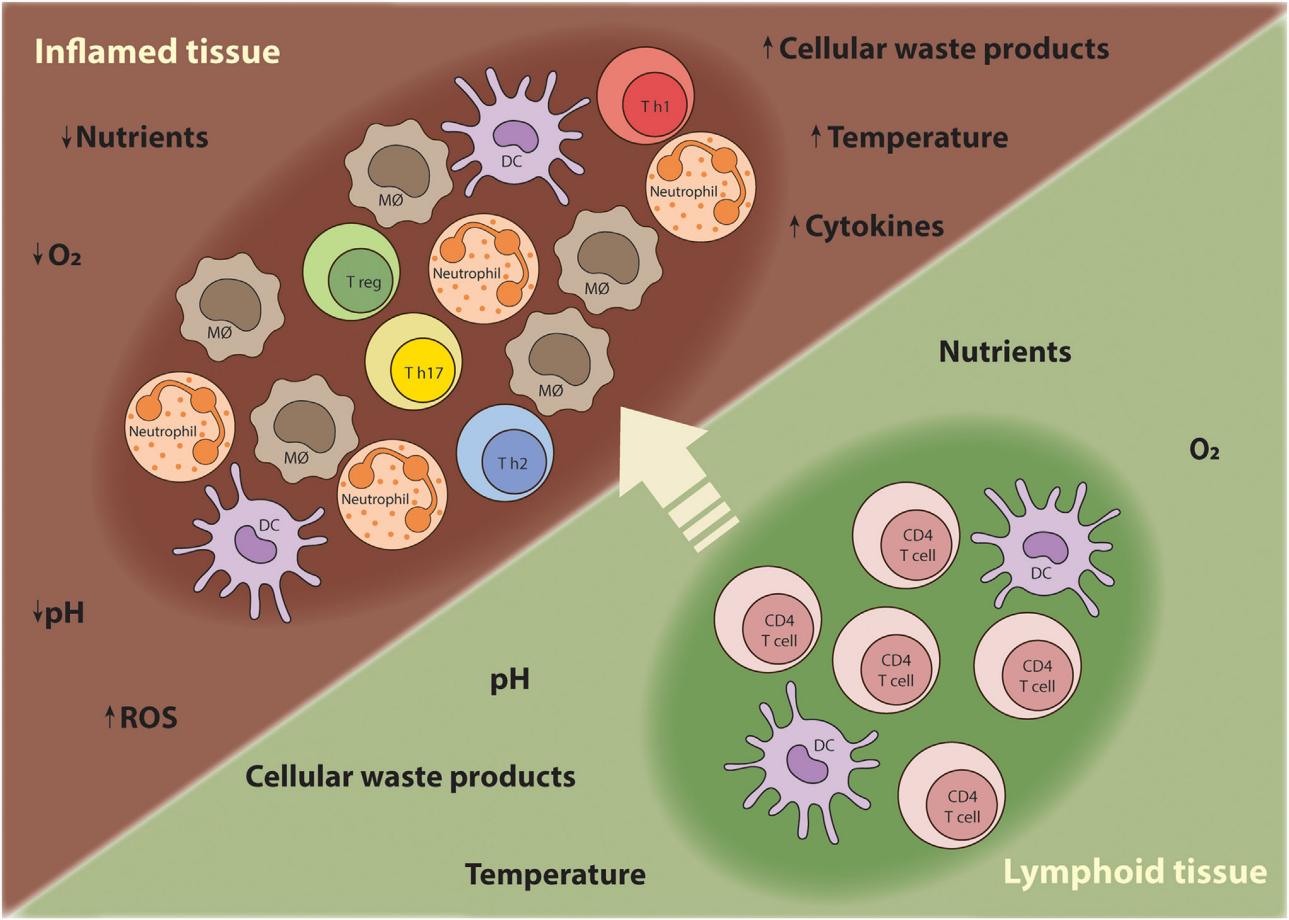
T cells require metabolic flexibility to adapt to the chronic inflammatory environment. As T cells mature during an immune response, they migrate from nutrient-rich secondary lymphoid tissues to sites of inflammation where nutrients, oxygen, and other growth promoting factors become limited. Moreover, other innate inflammatory cells are recruited to the inflamed tissue and compete for nutrients. Thus, T cells have to metabolically adapt to these harsh conditions in order survive, proliferate, and perform their effector functions.

Sites of inflammation are characterized by extensive recruitment of innate inflammatory cells and high proliferation rates of lymphocytes (8). The inflammatory responses often promote edema, which increases the distance between the parenchymal cells and blood vessels, creating a local microenvironment that is depleted of nutrients and oxygen (8). Thus, T cells traveling from nutrient-rich secondary lymphoid tissues to sites of inflammation have to adapt their metabolism to support anabolic growth and maintain their function at the low oxygen and nutrient levels characteristic of inflammatory lesions (Figure 1) (9). Although the characteristics of the chronic inflammatory site differ according to the tissue in which the disease unfolds, some shared features of inflammatory microenvironments include: low nutrient levels (glucose and glutamine); increased lactate production; decreased pH; and hypoxia and high concentration of reactive oxygen species (ROS) (10).

Inflammatory sites have long been described to harbor reduced glucose concentrations, which may be partly caused by proliferation of recruited leukocytes and invading pathogens (11–13). Activated T cells upregulate glucose metabolism to fuel macromolecular synthesis pathways and promote proliferation (3, 14, 15). Indeed, glycolysis is essential for T cell division, as T cells have decreased proliferation rates in glucose-deficient media, even in the presence of high levels of alternative energy sources like glutamine (16). Proliferating leukocytes are also ravenous glutamine consumers (17) and, although few studies have examined glutamine concentrations at inflamed sites, it seems likely that glutamine would decrease in the same manner as glucose. For example, in septic patients, plasma and skeletal muscle glutamine levels are decreased compared with healthy controls, and low glutamine concentrations are associated with poor prognosis (18–20). In addition, in a small study of patients receiving artificial nutrition, those with elevated markers of inflammatory stress had significantly lower concentrations of glutamine in plasma and gut mucosa (21). Similarly, patients with Crohn’s disease have lower levels of glutamine in inflamed mucosal tissues compared with non-inflamed mucosal tissues (22). Therefore, metabolic flexibility may be necessary to sustain T cell proliferation and effector function in chronic inflammatory environments.

Physiological lactate concentration in healthy tissues or blood is normally kept at 1.5–3 mM, but this can increase to as much as 10 mM in inflammatory environments such as atheroscle-rotic plaques or rheumatic synovial fluid (23). Lactic acid is a by-product of glucose metabolism and lactate accumulation can also be used as an indirect reporter of another inflammatory hallmark, decreased extracellular pH (24). Extracellular lactate and acidic conditions (low pH) have been shown to reduce the proliferation and function of human and mouse cytotoxic T cells due to decreased activation and inhibition of glycolysis (25–28), while restoration of pH to physiological levels rescues T cell function (25, 29). However, a recent study reported that CD4^+^ T cells sense lactate *via* the SLC5A12 transporter, and this interaction inhibits T cell motility, which might lead to T cells becoming entrapped at inflammatory sites, where they perpetuate the chronic inflammatory process (23).

Reactive oxygen species are key signaling molecules that play diverse roles in cellular function including cell signaling, differentiation, proliferation, and apoptosis. However, at high concentrations, they can act as mediators of inflammation due to their capacity to oxidize cellular constituents and damage DNA (30). Most ROS are generated as by-products of cellular metabolism *via* the electron transport chain (ETC), through partial reduction of the oxygen molecule during oxidative phosphorylation (OXPHOS) in mitochondria. Superoxide anion $\left(O_{2}^{•-}\right)$, the hydroxyl radical (^∙^OH), and hydrogen peroxide (H_2_O_2_) can all be generated in this way (31). ROS are abundant at inflammatory sites (32) and affect T cell functions (33, 34). For example, the presence of high levels of ROS in the environment has been reported to favor CD4^+^ T cell differentiation toward a Th2 phenotype, but the mechanisms involved remain unclear (35, 36).

Tissue hypoxia is characteristic of various chronic inflammatory diseases such as atherosclerosis, RA, and IBD, with oxygen levels considerably lower (<2%, equivalent to 2.026 kPa) than in healthy tissues (<5%, equivalent to 5.056 kPa) (37). However, even in healthy tissues, T cells can be exposed to varying oxygen concentrations ranging between 3 and 19% (3.039–19.247 kPa) as they migrate between blood and different tissues (38). The upper airways have the highest oxygen concentration (39), while lymphoid tissues have markedly lower oxygen concentrations; e.g., 6.5% (equivalent to 6.585 kPa) in bone marrow (40) and 3–4% (3.039–4.052 kPa) in the spleen (41, 42). Several studies have shown that CD4^+^ T cells have a reduced rate of proliferation and survival under hypoxic conditions (37, 43). When exposed to hypoxic environments, T cells upregulate the oxygen-sensitive transcription factor, hypoxia-inducible factor (HIF)-1α. HIF-1α modulates T cell differentiation and meta-bolism by promoting anaerobic glycolysis through increased expression of the glucose transporter Glut1, as well as induction of several glycolytic enzymes (4, 44, 45). Of note, in activated T cells HIF-1α can also be upregulated under normoxia to sustain the expression of glycolytic enzymes during Th cell differen-tiation (4, 46, 47).

Increasing sodium conditions *in vitro* by approximately 40 mM boosts T cell proliferation (48). In addition, secondary lymphoid tissues have higher osmolality than serum, suggesting that a high-salt environment *in vivo* favors T cell proliferation (49). There is some evidence to suggest that inflamed tissues could harbor high levels of salt. For example, excessive salt intake has been associated with enhanced induction of experimental autoimmune encephalomyelitis in mice (50, 51), worsening of disease activity in multiple sclerosis patients (52) and exacerbation of tissue damage in cardiovascular disease (53). Recent evidence suggests that high-salt environments favor T cell skewing toward a Th17 pro-inflammatory phenotype and impairs the suppressive functions of regulatory T (Treg) cells (50, 51, 54). Moreover, dietary supplementation with NaCl in a mouse model of graft-versus-host disease (GVHD) inhibited Treg function and aggravated clinical outcomes (54). Although these studies suggest that reducing salt concentrations could be beneficial for limiting pathological T cell responses in inflamed tissues, there are circumstances where reducing tissue salt concentrations may have deleterious effects. For example, a recent study found that regional hypersalinity in the renal medulla drives the recruitment and antibacterial functions of mononuclear phagocytes that prevent urinary tract infections spreading to the kidney (55). Moreover, further studies are required to determine the impact of high-salt environments on T cell metabolic processes.

The temperature gradients across the body are affected by inflammation in different ways. While internal organs such as the spleen and gut are subject to fluctuations of core body temperature during episodes of fever (37–39°C), the skin and muscles are subjected to a wider range of temperature gradients (29–37°C) (56). In addition, the normal core temperature of 37°C of both humans and mice oscillates throughout the day by approximately 1.7°C (57). Thus, lymphocytes circulating between these changing thermal compartments are required to function at various temperatures. The effects of hyperthermia on T cell function has been the subject of a few studies, and febrile temperatures are known to enhance T cell proliferation in response to mitogens (58, 59). More recently, febrile temperature was shown to induce changes in membrane fluidity in CD4^+^ T cells leading to macromolecular clusters that reduced the requirement for CD28 costimulation (60). Presently, little is known about whether the local increase in temperature during inflammation alters T cell metabolism. Of note, mice are generally housed at a temperature comfortable for clothed humans, 19–22°C, but the thermoneutral zone for mice is around 30–32°C (61). Some studies argue that mice housed under laboratory conditions are chronically cold-stressed and have a different metabolic and thermal phenotype than mice raised at thermoneutrality (62, 63). Thus, housing temperature of mice may be a variable that requires more consideration in immunometabolism studies.

Next to daily oscillations of core body temperature, other daily rhythms can influence immune cell function. Circadian rhythms, the body’s autonomous internal clock based on intricate transcriptional and translational feedback loops, anticipate and allow organisms to adapt to environmental changes by controlling a wide array of physiological and metabolic processes (64). Lifestyles that disrupt the inherent biological clock, such as shift work, have been associated with increased systemic levels of inflammatory markers (65, 66) as well as increased incidence of cardiovascular disease (67), metabolic disorders (68, 69), and cancer (70, 71). Interestingly, trafficking and migration of immune cells, including T cells, is also regulated by circadian rhythms (72) although the exact impact of these fluctuations on T cell function remains to be fully elucidated (73, 74). The circadian clock can also influence feeding schedules and therefore could indirectly affect the availability of nutrients (69, 75). For example, the levels of several intracellular micronutrients, including magnesium, have been shown to fluctuate rhythmically in two eukaryotic cell lines (76). As ATP needs to be bound to magnesium to elicit its biological function, fluctuations in intracellular magnesium levels could affect all cell processes that require ATP for energy (77). Moreover, manipulation of magnesium levels also leads to changes of the circadian period suggesting that magnesium acts as a “meta-regulator” of the cellular clock (76). Indeed, it has been proposed that the mechanistic target of rapamycin (mTOR) pathway, which controls protein synthesis associated with proliferative signals, is highly sensitive to Mg-ATP fluctuations (76, 78, 79). Nevertheless, whether daily fluctuations in availability of magnesium influence T cell function remains to be established.

## Metabolic Reprogramming in CD4^+^ T Cell Activation

**Figure 2:**
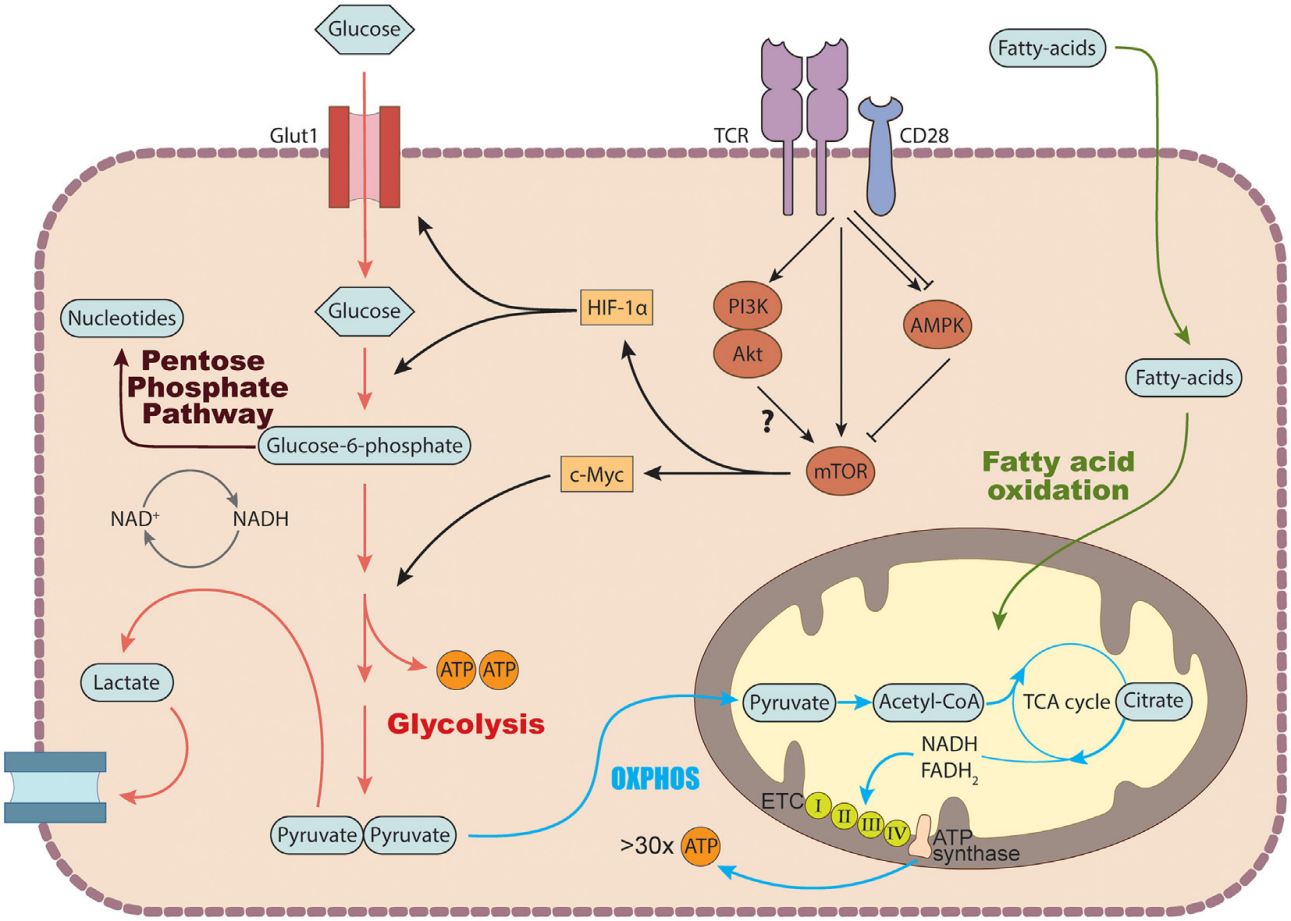
Basic metabolic pathways in T cells. In the cytosol, glucose is converted into pyruvate *via* glycolysis. Pyruvate can either be further metabolized into lactate and secreted or enter the tricarboxylic acid (TCA) cycle to generate NADH and FADH2 which get fed into the electron transport chain (ETC) to generate ATP *via* the process of oxidative phosphorylation. Glucose breakdown intermediates produced during glycolysis can be metabolized *via* the pentose phosphate pathway donating important building blocks for nucleotide and amino acid synthesis. TCR stimulation induces the expression of several genes involved in glucose transport and metabolism by recruiting the transcription factors c-Myc and hypoxia inducible factor (HIF-1) α *via* activation of the mechanistic target of rapamycin (mTOR) machinery. In addition to glucose, T cells can also use fatty acids as a source of energy by degrading fatty acids through fatty acid oxidation.

To drive the proliferation and differentiation of the appropriate leukocyte subsets needed to combat pathogenic infection, the immune system engages a series of coordinated growth and proli-ferative signals, including signals that modulate cellular metabolic processes (9) (Figure 2). Naïve, quiescent CD4^+^ T cells are characterized by a metabolic program that favors energy production over biosynthesis and generally rely on mitochondrial oxidative pathways, fueled by fatty acid or amino acid oxidation (80). Activation of CD4^+^ T cells triggers a dynamic network of transcriptional and translational changes which go hand in hand with metabolic adaptations to match the bioenergetic demands of the proliferating cells (80). During the initial phase after activation, oxidative metabolism is downregulated, while biosynthetic pathways are increased (81).

CD28 is one of the best characterized costimulatory receptors on naïve T cells, it promotes cell proliferation by activating different signaling pathways that sustain the bioenergetic demands associated with T cell activation (82). For example, CD28 costi-mulation has been described to upregulate glucose utilization allowing T cells to meet the biosynthetic demands associated with activation (83, 84). Furthermore, metabolic alterations driven by CD28 costimulation were recently shown to be important for recall of CD8^+^ T cell memory (T_m_) responses (85). The authors found that T cells primed *in vitro* with CD28 had tighter mitochondrial cristae (inner mitochondrial folds) and used the mitochondrial fatty acid oxidation (FAO) pathway significantly more than T cells primed without CD28 (85). Using an adoptive transfer model, they showed that T cells primed without CD28 were unable to prevent tumor outgrowth, suggesting that CD8^+^ T_m_ cell responses were impaired (85).

One critical signaling pathway for the transduction of the TCR/CD28 signal in activated T cells is the PI3K–Akt pathway, which promotes entry into the cell cycle and resistance to apoptosis; however, its role in the metabolic reprogramming of T cells is debated, since the requirement for Akt activation in promoting glycolytic metabolism in activated T cells is unclear (86). T cell activation also leads to the induction of the mechanistic target of rapamycin (mTOR) pathway, either through the PI3K–Akt pathway or independently. mTOR is a conserved serine/threonine kinase that integrates signals from various stimuli, including growth factors, glucose, amino acids, and oxygen levels, to regulate growth, survival, and proliferation (87) (Figure 2). In mammals, mTOR forms two functionally distinct complexes, mTORC1 and mTORC2, each with specific downstream targets and functions (87). mTORC1 is characterized by the presence of the scaffolding protein Raptor (regulatory-associated protein of mTOR), while mTORC2 is characterized by the presence of the Rictor (Rapamycin-insensitive companion of mTOR) protein (87). mTOR plays a pivotal role in regulating cellular metabolic pathways such as glycolysis, lipid synthesis, and amino acid meta-bolism.

During T cell activation, the glycolytic program is activated by transcription factors, such as c-Myc and HIF-1α, which orchestrate the expression of glycolytic enzymes and glucose transporters that facilitate increased uptake and catabolism of glucose (87). Once in the cytoplasm, glucose can be metabolized to yield two units of pyruvate, which are further processed according to the availability of oxygen. In aerobic conditions, pyruvate is further degraded to the acetyl group of acetyl-CoA and fed into the citric acid cycle, also known as the tricarboxylic acid (TCA) or Krebs cycle, which occurs in the inner layer of the mitochondria (88). The NADH and FADH_2_ produced at each turn of the cycle donate electrons to the ETC, a series of membrane-bound carriers, called complexes I–IV, located in the inner mitochondrial membrane (Figure 2). The large amount of energy released during the course of the electron transfer fuels the production of over 30 molecules of ATP through OXPHOS (88, 89).

By contrast, under low oxygen conditions, pyruvate is reduced to lactate *via* glycolysis, generating only two molecules of ATP per one unit of glucose (90). However, some cells, including activated T cells, convert glucose to lactate even when oxygen is not limiting. This aerobic glycolysis was first described by the German biochemist Otto Warburg for tumor cells, and it is thus known as the “Warburg effect” (91). Quiescent T cells generate most of their energy through OXPHOS, but T cell activation induces metabolic remodeling toward a program of anabolic growth and enhanced protein synthesis, necessitating greater uptake of nutrients. T cell activation is not merely a switch from OXPHOS to aerobic glycolysis, indeed both pathways are upregulated and cooperate to meet energetic demands, but glycolysis undergoes a marked increase and becomes the dominant metabolic pathway (90). Since ATP production *via* aerobic glycolysis is much less efficient than *via* OXPHOS, it seems counterintuitive that this pathway would dominate in proliferating T cells. However, increased glycolytic flux leads to increased production of NADH, which is used as a cofactor by numerous metabolic enzymes, as well as increased levels of glycolysis intermediates that are directed into anabolic pathways for the production of nucleotides, fatty acids, and amino acid precursors (92).

The amino acid glutamine is another critical substrate used by T cells during activation (17). Following stimulation, glutaminolytic enzymes and the glutamine antiporter CD98 are induced in a Myc-dependent fashion (93), and elimination of glutamine from the culture media decreases lymphocyte proliferation (94, 95). Glutamine is metabolized by glutaminolysis, and the intermediates produced in this process can enter the TCA cycle (17). Glutamine also acts as a nitrogen donor for the synthesis of purine and pyrimidines and is therefore able to facilitate the synthesis of nucleotides during cell proliferation (96).

Under harsh environmental conditions, cell survival is also determined by the capacity to recycle cellular nutrients as well as sense extracellular stimuli. The mTOR and adenosine monophosphate-activated protein kinase (AMPK) pathways play important roles in tailoring the metabolic adaptations of CD4^+^ T cell subsets during conditions where nutrients and oxygen are scarce. AMPK is a serine/threonine kinase that modulates cellular energy status in response to nutrient variations or physiological stress (97). Increases in cytoplasmic AMP-to-ATP concentrations activate the AMPK sensor. When activated, AMPK initiates metabolic reprogramming by switching on ATP-producing pathways (oxidation of glucose and fatty acids) and switching off ATP-consuming anabolic pathways (fatty acid or protein synthesis) (98). AMPK can also activate autophagy, directly or indirectly, to initiate metabolic reprogramming toward catabolic reactions (99). Autophagy is a cellular process in which the cell breaks down large cytoplasmic components such as organelles (also known as macroautophagy) to ensure sufficient metabolites when nutrients are low (100). AMPK can directly phosphorylate the autophagy proteins Ulk1/2 at multiple sites (99) and can also activate autophagy indirectly through suppression of mTORC1 signaling (99).

## How Nutrient Availability Shapes CD4^+^ T Cell Metabolism and Immune Responses

The balance between protective immunity and chronic inflammation requires that T cells appropriately differentiate into the effector or regulatory lineages. In addition to multiple cues from the microenvironment, such as the presence of key cytokines, distinct metabolic programs also support the differentiation of CD4^+^ T cells into their separate functional subsets (2). For example, it is largely accepted that Th1, Th17, and Th2 effector cells utilize higher rates of glycolysis, while Tregs preferentially use FAO (3). In addition, as immune responses are terminated, most effector CD4^+^ T cells undergo apoptosis, but some become memory T cells and revert back to OXPHOS and FAO. These memory T cells provide long-term protective immunity and do not rapidly proliferate in the absence of antigen rechallenge, and thus do not require high rates of glycolytic metabolism (2).

### Effector T Cell Responses Are Dynamic Processes

#### Th1 Cell Function Is Highly Dependent on Environmental Levels of Nutrients

Th1 cells are considered highly glycolytic and glutaminolytic cells and rely on these pathways to support their growth and proliferation (3). Depriving naïve CD4^+^ T cells of glutamine during TCR stimulation results in generation of Foxp3^+^ Treg cells, even in the presence of Th1-polarizing conditions (101). The breakdown product of glutaminolysis, α-ketoglutarate, may act as a metabolic regulator for Th1 differentiation and prolifera-tion by promoting the expression of T-bet and enhancing mTORC1 signaling (101). Therefore, although environmental depletion of glutamine might promote resolution of inflammation by favoring Treg cells, under other circumstances impaired Th1 responses could enhance pathogen spread, leading to aggravated tissue damage.

T cell activation and differentiation also depend on the availability of other amino acids. For example, LAT1, an L-type amino acid transporter responsible for the uptake of phenylalanine, tyrosine, leucine, arginine, and tryptophan, is required for proliferation and differentiation into Th1 and Th17 cells *in vitro* (102). Furthermore, a recent study linked the complement system to regulation of amino acid and glucose uptake in human Th1 cells (103). Signaling through CD46, a key costimulatory molecule and complement regulator expressed on human CD4^+^ T cells, was important for effective Th1 cell differentiation by potentiating TCR-driven Glut1 expression. Mechanistically, TCR/CD28 signaling induced the production of complement C3b that activated CD46 in an autocrine manner, resulting in the nuclear translocation of its cytoplasmic tail isoform CYT-1 (103). Nuclear CYT-1 induced transcription of LAMTOR5, which mediated upregulation of Glut1 and LAT1 and also activated mTORC1. These events were crucial for human Th1 cell differentiation as siRNA knockdown of CD46 or LAMTOR5 resulted in selective suppression of Th1 cells (103).

Glucose uptake and aerobic glycolysis is essential for IFNγ production in Th1 cells (104–106). Two mechanisms have been put forward to explain this observation. Chang et al. reported that the cytokine production is limited by the binding of the glycolytic enzyme GAPDH to the 3′ UTR of *ifng* mRNA, but this inhibition is diminished when GAPDH is engaged in its enzymatic function during glycolysis (104). More recently, Peng et al. proposed an epigenetic mechanism through which glycolysis promotes IFNγ production in Th1 cells (106). They found that expression of lactate dehydrogenase A (LDHA) in activated T cells was required to sustain aerobic glycolysis and support Th1 differentiation and that this was not dependent on the *ifng* 3′ UTR (106). Instead, LDHA-deficient T cells had severely reduced histone H3K9 acety-lation (a marker associated with active transcription) at the *ifng* locus (106). Mechanistically, deletion of LDHA abrogated lactate production, thus shunting pyruvate into the mitochondria, which enhanced OXPHOS, but reduced citrate export out of the mitochondria leading to decreased cytosolic acetyl-CoA levels—the critical substrate needed for histone acetylation of gene loci through histone acetyltransferase (106). The importance of this pathway for Th1 responses *in vivo* was shown by their observations that conditional deletion of LDHA in T cells protected susceptible mice from Th1-mediated lethal auto-inflammatory disease (106). These studies suggest that when Th1 cells migrate to a glucose-deprived inflammatory environment, the reduced glucose availability will lead to a drop in glycolysis and decreased IFNγ production, possibly representing intrinsic negative feedback mechanisms to inhibit excessive Th1-mediated immune pathology.

#### Cellular Lipid Metabolism Supports Th17 Differentiation and Function

Th17 cells are largely confined to barrier sites such as the intestine, lungs, or skin, where they play a key role in defense from opportunistic pathogens and maintenance of epithelial barrier function (107, 108). However, it is also evident that their aberrant production of inflammatory cytokines is important in driving a number of autoimmune diseases (109, 110). Recent evidence suggests that lipid metabolic pathways play a role in regulating the dichotomous function of Th17 cells under normal and pathogenic conditions (111–113).

Lipogenic pathways are a crucial part of T cell metabolic repro-gramming, as proliferating T cells require fatty acids for membrane synthesis and also for a plethora of other cellular processes, such as signaling and energy production. Activated T cells rapidly augment fatty acid synthesis (FAS) while concomitantly decreasing FAO (93). Of note, free fatty acids have been found to be highly enriched in the inflamed tissues of conditions associated with excess fat deposits, such as obesity and atherosclerosis (10). FAS takes place in the cytosol and commences with ATP consumption through the carboxylation of acetyl-CoA to malonyl-CoA, a reaction catalyzed by acetyl-CoA carboxylase 1 (ACC1) (114). By contrast, FAO occurs mainly in the mitochondria and involves generation of acetyl-CoA, which can be directly shuttled into the TCA cycle and further oxidized to generate ATP *via* OXPHOS. The enzyme acetyl-CoA carboxylase 2 is located in the inner mitochondrial membrane and promotes mitochondrial FAO (111).

These fatty acid metabolic pathways have emerged as important regulators of Th17 function. ACC1 can regulate the balance between Th17 and Treg cells, as pharmacological or genetic block-ade of ACC1 impaired differentiation of human and mouse Th17 cells but favored the induction of Foxp3^+^ Treg cells (111). The authors proposed that Th17 cells use ACC1-driven FAS to produce phospholipids for cellular membranes, while Treg cells actively take up exogenous fatty acids to sustain their proliferation (111). Consistent with these results, in a GVHD model, mice adoptively transferred with ACC1-deficient T cells showed reduced mortality and also higher frequencies of Treg cells in the colon in comparison with mice that received WT T cells (115). In addition, the intracellular levels of different fatty acid species may affect the pathogenicity of Th17 cells by modulating their cytokine responses. Single-cell RNA-sequencing of Th17 cells generated under pathogenic or non-pathogenic polarizing conditions implicated expression of *Cd5l*, encoding CD5 antigen-like (CD5L) protein (also known as AIM), as a regulator of Th17 cell pathogenicity (112). CD5L is a member of the scavenger receptor cysteine-rich superfamily involved in lipid metabolism, specifically in inhibition of fatty acid synthase (116). Although the mechanism remains incompletely understood, CD5L seemed to alter the intracellular balance between polyunsaturated and saturated fatty acids, thus affecting the function of two metabolic genes—*cyp51* and *sc4mol*—that synthesize ligands for RORγt, a Th17 master transcription factor (112, 113). This in turn might lead to increased RORγt binding at the anti-inflammatory genes (*IL-10)* and reduced binding at the *IL-17* and *IL-23r* loci (pro-inflammatory genes) in Th17 cells (113).

Another cell-intrinsic metabolic pathway that has been associated with a pathogenic Th17 phenotype is mTORC1. Sasaki et al. reported that deletion of *p70S6KI* (which encodes a serine/threonine kinase that is downstream from mTORC1) resulted in decreased expression of Th17-associated genes, including *il17a, il17f*, and *il23r* (117). By contrast, differentiation into Treg, Th1, or Th2 cells was not altered in the absence of *p70S6KI*, suggesting that it plays a selective role in the differentiation of Th17 cells (117). Consistent with these findings, *p70S6KI*-knockout mice exhibited delayed development of EAE (117).

Given the low oxygen availability at inflammatory sites, it is perhaps unsurprising that HIF-1α also plays a crucial role in adapting the T cell metabolic program to the hypoxic conditions and skewing the balance between Th17 inflammatory cells and Treg immunosuppressive cells. HIF-1α promotes glycolysis and increases the expression of RORγt while targeting Foxp3 for proteasomal degradation (4, 46). Indeed, deletion of HIF-1α in CD4^+^ T cells abrogated Th17 development and promoted Treg cell differentiation, even under Th17 culture conditions (4, 46). Further evidence that the hypoxic environment influences Th17 function comes from a study in which human Th17 cells differentiated *in vitro* under hypoxic conditions (1% O_2_) had increased secretion of the anti-inflammatory cytokine IL-10 (118).

Furthermore, another recent study reported that *in vitro* differentiated Th17 cells use both OXPHOS and glycolysis, whereas Th17 cells isolated from steady state and inflamed tissues rely on OXPHOS to generate the energy required for cytokine production (119). Consistent with these findings, administration of the OXPHOS inhibitor oligomycin reduced inflammatory Th17 cytokine production *in vivo* and decreased pathology in a mouse model of colitis (119). Higher expression of pyruvate dehydrogenase kinase 1 (PDK1) by *in vitro*-generated Th17 cells correlated with their enhanced glycolytic metabolism (119), consistent with a previous report that PDK1 was essential for Th17 differentiation *in vitro* (120). *In vitro* differentiation of T-helper cell subsets is widely used to generate large numbers of effector cells for analyses. However, these findings regarding Th17 cells indicate that the *in vitro* differentiation conditions may affect the metabolic phenotype of the cells, which in turn could endow them with slightly different functional characteristics than their *in vivo* counterparts.

#### Th2 and Th9 Cells Depend on mTOR Function and Glycolytic Metabolism

Several inflammatory pathologies are associated with a Th2 cell component, including diseases associated with IgE and type 2 cytokine secretion (IL-4, IL-5, and IL-13), such as allergy, chronic asthma, and atopic dermatitis (121).

Physiological type 2 immune responses seem associated with an oxidative metabolism, as Th2 cytokines (IL-4 and IL-13) activated an STAT6-dependent program of oxidative metabolism involving peroxisome proliferator activated receptors γ and δ (PPARγ and PPARδ) in macrophages (122). Interestingly, several recent studies have also implicated PPARγ in effector Th2 function (123–125). For example, activation of the TCR/CD28-mTORC1 pathway facilitated complete activation and proliferation of Th1 and Th2 cells by promoting fatty acid uptake through increased expression of PPARγ (123). In addition, PPARγ was critical for Th2 cell responses to house dust mite and *Heligmosomoides polygyrus* antigens, as mice lacking PPARγ failed to generate IL-5- and IL-13-producing Th2 cells (124, 125). Mechanistically, PPARγ was necessary for the upregulation of the IL-33 receptor on differentiating Th2 cells in the lung, thereby promoting full Th2 effector responses. This may suggest that oxidative metabolism may have a role in the effector function of Th2 cells and promotion of pathogenic responses. However, it is important to point out that *in vitro*-polarized murine Th2 cells exhibited high glycolytic rates, similar to Th1 and Th17 cells (3, 4).

Although mTORC1 signaling is needed for Th2 lineage commitment (126), multiple studies support a role for mTORC2 as a preferential signaling pathway in the differentiation, function, and metabolism of Th2 cells (127, 128). For example, Rheb-deficient mice have impaired mTORC1 function and fail to generate Th1 and Th17 cells but are able to differentiate Th2 cells (128). Conversely, T cells from Rictor-deficient mice, in which mTORC2 activation is impaired, fail to differentiate into Th2 cells but are able to generate Th1 and Th17 cells (128). In addition, SGK1, another downstream target of mTORC2, was shown to promote commitment to the Th2 cell lineage while simultaneously blocking differentiation into the Th1 lineage (129). SGK1 prevents degradation of JunB (129), which was previously described as a Th2 cell-specific transcription factor that regulates the Th2 cytokine program (130). Moreover, genetic deletion of the GTPase RhoA, a downstream target of mTORC2, decreased glycolysis and impaired IL-4 production in murine Th2 cells, and protected mice against airway inflammation in an OVA-induced model of allergic asthma (131). Consistent with these observations, a recent genome-wide transcriptional profiling study of human Th2 cells isolated from allergic asthma patients found a positive correlation between c-Myc expression and disease status (132), again pointing toward glycolysis as a marker of Th2 cell pathogenicity. Although it seems difficult to reconcile these reports with the studies above that implicated FAO in Th2 function, it may be that metabolic flexibility is important for optimal differentiation and function of Th2 cells. Thus, while mTORC2-driven glycolysis might be primarily required for differentiation and proliferation of Th2 cells, a mixed metabolic profile, incorporating both FAO and glycolytic activity, may be important to sustain effector Th2 cells in peripheral tissues.

Th9, characterized by the production of IL-9, are closely linked to Th2 cells (133). Th9 effector cells develop from naïve CD4^+^ T cells in the presence transforming growth factor-β (TGF-β) and IL-4, secrete IL-9 in large amounts, are also known to produce IL-10 and IL-21, and have been implicated in some inflammatory allergic processes, such as asthma (134). A recent study shed some light on the metabolic properties of Th9 cells. Wang et al. reported that *in vitro* differentiated Th9 cells were highly glycolytic in comparison with Th1, Th2, Th17, or Treg cells (47) and identified SIRT1 (sirtuin-1) as a negative regulator of Th9 cells. SIRT1 is an NAD^+^-dependent enzyme that can deacetylate histone residues on chromatin, but it has also been proposed to act as an NAD^+^-dependent metabolic sensor (135). SIRT1 was shown to inhibit Th9 cell differentiation *in vitro* and mice harboring a T cell-specific deletion of SIRT1 exhibited exacerbated airway inflammation in an OVA-induced allergy model (47). Further investigations linked TAK1 (TGF-β activated kinase), an important mediator of TGFβ signaling, with active suppression of SIRT1 in Th9 cells. The study proposed that TAK1 suppression of SIRT1, coupled with an increased mTORC1-driven glycolytic metabolism, was crucial for Th9 cell differentiation (47).

#### T Follicular Helper (Tfh) Cells Are Metabolically Adapted to the Germinal-Center (GC) Environment

T follicular helper cells are characterized by the expression of the chemokine receptor CXCR5, the inducible T cell costimulator ICOS, the transcription factor Bcl-6, and the production of IL-21 (136). They have a critical role in the formation and maintenance of GCs that promote the generation of affinity matured B cells (136). Several studies have linked dysregulated Tfh cell responses with autoimmune disease (137), and inflammatory sites often develop lymphoid aggregates, termed ectopic lymphoid structures (ELS), which act like functional GCs and comprise B cells and Tfh-like cells (138). The conditions within the ELS present at the inflammatory sites remain unknown; however, one could speculate that they would resemble those present in GCs.

German centers are restricted microenvironments where antigen-activated B cells undergo clonal expansion and introduce point mutations into the hypervariable regions of the BCR genes to allow for affinity maturation (139). GCs are organized into two distinct zones (termed light and dark) and B cells repeatedly cycle through the zones as they mature (139). The dark zone is associated with rapid B cell proliferation, while the light zone is traditionally associated with B cell affinity maturation and class switching, requiring help from Tfh cells (136). Cho et al. showed that mouse GC light zones are hypoxic environments with increased levels of HIFs which limit the proliferation and survival of GC B cells (140). Although this hypoxic environment may seem detrimental for B cell development, it may act as a threshold for B cell selection, reducing the risk of abnormal B lymphocyte development (140). An independent study confirmed the hypoxic nature of the GC microenvironment and showed that reversing hypoxia by placing mice in chambers containing 60% O_2_ resulted in a decreased frequency of Tfh cells, reduced GC formation and impaired class switching following immunization (141). Thus, the hypoxic environment sustains the GC reaction and positively impacts on Tfh function.

Moreover, given that GCs are sites of constant proliferation it is likely that the availability of glucose and of other nutrients might be limited. Precisely how Tfh cells adapt to the metabolic demands of the GC microenvironment is not completely understood, but some studies have postulated that a restriction in mTORC1 activation may be involved. It was reported that hypoxia inhibits mTORC1 activation in GC B lymphoblasts through an HIF-1-dependent mechanism that limits the expression of amino acid transporters (140). Whether the same mechanism operates in Tfh cells is unclear, but decreased mTORC1 activity was reported to favor Tfh cell differentiation at the expense of Th1 and T-bet expression (142). Using an acute viral infection model, Ray et al. showed that shRNA silencing of mTOR or Raptor promoted Tfh cell differentiation, while silencing of Rictor had minimal effects on Tfh cells and instead promoted Th1 development (142). In addition, they found that Tfh cells were less glycolytic than Th1 cells and instead relied mainly on mitochondrial respiration (142). The authors suggested that this might be driven by Bcl-6, as overexpression of Bcl-6 in naïve CD4^+^ T cells recapitulated the metabolic characteristics observed in Tfh cells (142). This suggestion is consistent with previous reports in which Bcl-6 was described to downregulated genes associated with glycolysis (143, 144). Overall, these findings suggested that inhibition of mTORC1 signaling and glycolytic metabolism play an important role in the Tfh cell adaptation to the GC environment. However, recent studies using a CD4-Cre approach to drive T cell-specific deletion of Raptor or Rictor highlighted a requirement for both mTORC1 and mTORC2 in Tfh differentiation (145, 146). Thus, deletion of Raptor (mTORC1) or Rictor (mTORC2) in T cells led to decreased GC formation and Tfh differentiation upon antigen immunization or viral challenge (145, 146). Furthermore, Zeng et al. observed that *in vitro* differentiated Tfh-like cells expressed elevated levels of Glut1 in comparison with activated non-Tfh cells. Similarly, Glut1 expression was higher on Tfh cells than non-Tfh cells isolated from Peyer’s patches (PP), both at steady state and upon foreign antigen challenge (146). Given that mTORC1 is required for T cell quiescence exit, Zeng et al. also explored the effects of conditional deletion of Raptor or Rictor in mature peripheral CD4^+^ T cells using an OX40-Cre driver. They found that conditional deletion of Raptor or Rictor in activated CD4^+^ T cells led to severely reduced GC formation and Tfh cell responses in PP, both at steady state and upon viral infection (146). Thus, these studies argue for positive and non-redundant roles for mTORC1 and mTORC2 in Tfh cell differentiation.

The contradicting results reporting positive or negative effects of mTORC1 and mTORC2 activation in Tfh responses are difficult to reconcile, but some of the differences might be due to the approaches used to delete or silence components of the mTOR-signaling pathway, as well as the potential differences between *in vitro* or *in vivo* generated Tfh cells. It may be that while both mTORC1 and mTORC2 are required for Tfh generation, tempering the levels of mTORC1 activation may facilitate optimal Tfh responses in GC.

### Foxp3^+^ Treg Cells Are Endowed With Metabolic Flexibility

CD4^+^Foxp3^+^ Treg cells produce immunosuppressive cytokines such as IL-10 and TGF-β and are critical for maintaining immune tolerance and preventing deleterious inflammatory responses (147). Treg cells have distinct metabolic requirements and have been described to preferentially rely on OXPHOS driven by lipid oxidation, rather than glucose, for ATP production (3). However, recently it has been suggested that metabolic adaptations of Treg cells are context dependent and are influenced by factors such as Treg cell origin or anatomical distribution (148), although, it still remains largely undetermined how distinct subpopulations of Tregs, i.e., thymic derived (tTregs), peripherally induced (pTregs), and *in vitro* generated iTregs, differ metabolically.

The transcription factor Foxp3, which is indispensable for Treg development, function, and maintenance, was recently demonstrated to also play a major role in regulating their metabolism. It was found that Foxp3 expression was sufficient for the increased OXPHOS activity observed in mouse Foxp3^+^ iTreg cells generated *in vitro* and that Foxp3 increased expression of ETC protein complexes that may influence Treg suppressive abilities (149, 150). In addition, Foxp3 may decrease glycolysis by inhibiting c-Myc expression through binding to the TATA box of the *Myc* gene (149). In some circumstances, aberrant increases in glycolytic activity in Foxp3^+^ Treg cells have been associated with their dysfunction and consequent inflammation (151, 152). Murine iTregs express low levels of the glucose transporter Glut1 in comparison with effector T cells, but comparable levels to naïve T cells (3) and in human Treg cells, Glut1 expression is thought to be limited by Foxp3 through inhibition of Akt (153). Consistent with these findings, murine Treg cells overexpressing a transgenic Glut1 receptor had reduced CD25 and Foxp3 expression and could not suppress colitis in an adoptive transfer model (154).

However, it appears that increased mTORC1 activity and glycolytic metabolism might be necessary to ensure adequate Treg cell proliferation under inflammatory conditions. Evidence suggests that inflammatory stimuli and Foxp3 have opposing effects on Treg proliferation and function by differentially regulating mTORC1 and glucose metabolism. Thus, treatment of activated Treg cells with a TLR1/TLR2 agonist enhanced activation of mTORC1 and increased their proliferation but impaired their suppressive function (154). By contrast, Foxp3 expression in Treg inhibits mTORC1 signaling and glycolysis but promotes oxidative metabolism and slows their proliferation (154). These findings are consistent with previous studies showing that excessive mTORC1 activity impairs Treg function (1, 155) and with the recent discovery that Treg cells engage the serine–threonine phosphatase PP2A to suppress mTORC1 activity (156). However, other work reported that the induction and suppressive function of human iTreg cells, generated by suboptimal TCR stimulation, was tightly dependent on glycolysis (157). Mechanistically, the authors proposed that glycolysis controls the expression of the full-length *FOXP3* containing exon 2 splice variant (Foxp3-E2), responsible for the suppressive activity of Treg cells, through the glycolytic function of enolase-1 enzyme (157). Of note, human Tregs are highly proliferative *in vivo* but are hyporesponsive to TCR stimulation *in vitro* (158). Thus, it is likely that they engage glycolysis in certain contexts. Indeed, comparative proteomic analyses of human Treg and Tconv cells found that freshly isolated human Treg cells were highly glycolytic and proliferative, while *in vitro* proliferating human Tregs engaged glycolysis, but also FAO (158). In comparison, Tconv cells switched from OXPHOS to aerobic glycolysis upon *in vitro* activation (158). This again underscores that the metabolic phenotype of T cells is heavily influenced by their environmental differentiation conditions.

Overall, one could speculate that at the beginning of inflammatory process, when glucose is still available for proliferating T cells, Treg cells use glycolytic metabolism to increase their numbers, while during chronic inflammation when glucose is scarce their reliance on OXPHOS and FAO might enable them to perform suppressor functions in the effort to resolve the inflammatory process. In addition, it is also conceivable that the utilization of glucose by proliferating Treg cells could be another mechanism of immunosuppression, by depriving effector T cells of this nutrient.

#### Nutrient-Depleted Environments Support Immunosuppressive Responses

As detailed in Section “The Inflammatory Microenvironment,” the inflammatory site is characterized by high concentrations of lactate and low-glucose levels. Previous reports have shown that l-lactate strongly suppresses effector T cells (26, 29, 159) (Scharping) and that T cells do not proliferate in glucose-deficient media (16). However, a recent study suggested that high concentrations of l-lactate do not affect Treg proliferation or their suppressive function under conditions of reduced glucose supply (149). The authors proposed that the metabolic advantage of Tregs in high lactate environments could be based on resistance to NAD^+^ depletion. Teff cells rely on aerobic glycolysis to proliferate and reduce NAD^+^ to NADH during the breakdown of glucose to pyru-vate. Lactate dehydrogenase (LDH) further catalyzes the reduction of pyruvate into lactate (149). However, in a high lactate environment, LDH favors the reverse reaction, converting lactate to pyruvate while using NAD^+^ as a cofactor. Thus, Teff cells face a redox imbalance when insufficient NAD^+^ is present and glycolysis cannot proceed (149). By contrast, in Treg cells, Foxp3 inhibits glycolysis and promotes OXPHOS, which allows the cells to generate NAD^+^ by oxidation in the TCA cycle (149). In addition, another recent report showed that Tregs may have developed a preference for FAO metabolism to avoid fatty acid-induced cell death (150). Long-chain fatty acids, such as palmitate, are known to have proapoptotic effects through various mechanisms, including depolarization of the mitochondrial action potential and generation of ROS (160). This study showed that Foxp3 endows Treg cells with an increased ability to utilize fatty acids as fuel for OXPHOS by upregulating the enzymes involved in FAO, thus increasing resistance to long-chain fatty acid-induced apoptosis (150).

Regulatory T cells also seem to have enhanced resistance to amino acids deprivation. For example, depriving CD4^+^ T cells of glutamine during activation results in generation of Foxp3^+^ Treg cells, even in the presence of Th1-polarizing conditions (101). Similarly, deficiencies in the amino acid transporters Slc7a5 and Slc1a5 (ASCT2) impaired glutamine uptake and decreased Teff cell differentiation without affecting the generation of Treg cells (102, 161). Moreover, Treg cells can stimulate dendritic cells to express enzymes that catabolize essential amino acids, thus reducing their availability in the local microenvironment. Consequently, this limits Teff cell differentiation and further promotes the expression of Foxp3 cells by CD4^+^ T cells (162).

Thus, Tregs show a high degree of flexibility in fuel choice, and the ability to increase their OXPHOS capacity might represent a survival advantage in conditions of low nutrients. In addition, their adaptations to nutrient-deplete environments might give them a survival advantage at the inflammatory site and thus the ability to outlive the Teff cells and trigger the resolution of the inflammatory process.

#### Treg Cells Adapt to the Tissue Environment by Promoting Autophagy and Nutrient Recycling

It is also worth considering that inhibition of catabolic processes, such as autophagy, can alter the availability of nutrients thus leading to dysregulations in cell metabolism. Two recent studies described a crucial role for autophagy in regulating cellular metabolism of peripheral Treg cells. Kabat et al. generated mice in which the essential autophagy gene *Atg16l1* was selectively deleted in Foxp3^+^ Treg cells and found that these animals developed severe spontaneous multiorgan inflammation by 5 months of age (151). This was characterized by an accumulation of Th effector subsets and a drastic depletion of Foxp3^+^ Treg (151). Further analyses revealed that *Atg16l1*-deficient Treg cells had higher expression of glycolytic genes than Treg cells from control mice, whereas genes involved in FAS/FAO were markedly decreased (151). These metabolic differences were much more pronounced in Treg cells originating from the colonic lamina propria than from the spleen (151). Consistent with these findings, a parallel study reported that mice with selective deletion of the essential autophagy genes Atg7 or Atg5 in FoxP3^+^ Treg cells developed severe multiorgan inflammation by 5 months of age (152). *Atg7*-deficient Treg cells from these animals had aberrant mTORC1 activity, associated with increased c-Myc expression and heightened glycolytic metabolism (152). Although precisely how autophagy deficiency in Treg cell impairs their survival remains to be elucidated, these studies suggest that autophagy is important for Treg metabolic flexibility, particularly required for adaptations in peripheral tissues (151).

## Therapeutic Perspectives

As we have described so far, different CD4^+^ Th subsets are asso-ciated with overlapping but distinct metabolic profiles. This leads to the attractive hypothesis that targeting metabolic pathways could underpin new therapeutic strategies for immune and inflammatory diseases. Several studies have attempted to manipulate the effector function of lineage-committed T cells through interventions directed at metabolic pathways. For example, targeting PDK1 with dichloroacetate selectively inhibited the survival, function, and proliferation of Th17 cells and diminished inflammation in models of colitis and EAE (120). Similarly, blocking the ACC enzymes involved in *de novo* FAS with Soraphen A was shown to bias T cell differentiation away from Th17 cell development and toward a Treg fate (111). Therapeutic strategies like these could be exploited in autoimmune diseases with a strong Th17 component, such as multiple sclerosis. Also, depending on the setting, T cells may use distinct metabolic phenotypes to adapt to their environment, in nutrient scarce inflammatory environments, CD4^+^ T cells have to compete for nutrients with other leukocytes, as well as parenchymal and stromal cells. Therefore, another potential way to interfere with T cell metabolism is by blocking nutrient transporters on the cell surface. The solute-carrier (SLC) receptor superfamily are membrane-bound transporters that carry various molecules, including glucose and amino acids, across the cell membrane (163). Transporter families often have multiple isoforms with distinct substrate specificities and different expression levels between different cell populations, suggesting that developing a selective therapy that targets only a particular Th cell subset might be possible. For example, L-type amino acid transporter 1 (LAT1 or SLC7A5) is the main large neutral amino acid transport in activated T cells and genetic deletion of this transporter prevented the proliferation of CD4^+^ T cells, but their ability to differentiate into Treg cells was preserved (102). Moreover, pharmacological inhibitors of SLC7A5 were shown to constrain the inflammatory function of cytotoxic T cells (102).

Given the multitude of roles that the mTOR signaling pathways play in CD4^+^ T cell differentiation and function it is not surprising that mTOR inhibition has been sought as a potential therapy for chronic inflammatory conditions. The first known pharmacological inhibitor of mTOR, rapamycin, was originally developed as an immunosuppressive agent for organ transplant rejection, and mTOR was subsequently identified as its molecular target (164). The observations that the mTOR pathway differentially modulates Treg cells compared with effector Th1/Th17 cells provided additional rationale for pharmaceutical targeting of mTOR in human disease (1). In addition, overactivation of mTOR signaling through deletion of TSC1, an upstream negative regulator of mTOR, leads to reduced Treg suppressive activity in a mouse model of colitis (165). Another study showed that pharmacological inhibition of mTORC1 ameliorated DSS-induced colitis through reduced differentiation of both Th1 and Th17 cells (166). Consistent with these differential effects on Teff cells and Treg cells, mTORC1 inhibitors have been used as adjuncts during *ex vivo* expansion of human iTregs, to stabilize Foxp3 expression and function, while preventing outgrowth of Teff cells (167). These human iTregs maintained their suppressive activity after transfer into immunodeficient mice, suggesting that therapeutic Treg regimes may be enhanced by inhibiting mTORC1 (167).

It is clear that modulating metabolism *via* mTOR has potent effects on CD4^+^ T cells. However, mTOR plays a role in many different cells, and non-selective manipulation of this pathway has a high potential for deleterious side effects. Indeed, a wide range of adverse effects are associated with rapamycin treatment, including metabolic abnormalities (hyperlipidemia), skin reactions, and increased opportunistic infections (164).

Therapies aimed at other molecular targets within metabolic pathways are under development. Considering the capacity of AMPK to halt anabolic metabolism it is possible that targeting AMPK activation could lead to regulation of inflammatory T cell function. A few studies have already examined the effects of pharmacological activators of AMPK in T cells. Metformin is a widely used drug in the management of type 2 diabetes due to its benefits in relation to glucose metabolism (168). However, several studies have suggested additional beneficial roles for metformin in cardiovascular protection, cancer targeting, or aging (169). Although the mechanism of action of metformin is incompletely understood, it is thought that metformin suppresses glucose production in the liver. At the cellular level, it is largely accepted that metformin inhibits mitochondrial respiration, but how this is transduced to the health promoting effects of metformin remains unclear, and metformin has been described to act *via* both AMPK-dependent and AMPK-independent mechanisms (168). By blocking mitochondrial respiration, metformin prevents ATP production and thus leads to an increased cytoplasmic ratios of ADP:ATP and AMP:ATP, leading to activation of AMPK (170). Activated AMPK inhibits FAS and instead promotes FAO, reducing hepatic lipid stores and enhancing insulin sensitivity. It was recently shown that metformin’s mechanism of action relies on the nuclear pore complex (NPC) and the enzyme acyl-CoA dehydrogenase family member-10 (ACAD10) (171). By suppressing mitochondrial respiratory capacity, metformin reduces cellular ATP, restricting transit of the GTPase RagA/RagC heterodimer through the NPC, thus preventing activation of mTORC1. This subsequently enhances the transcriptional induction of ACAD10 and both the NPC and ACAD10 are required for the functional effects of metformin (171).

Treating mice with metformin has shown positive effects in several inflammatory disease models, including experimental autoimmune encephalomyelitis (172), IBD (173, 174), and GVHD (174). These studies associated metformin treatment *in vivo* with a reduction in Th17 cells and a rise in Treg cells (172–174). However, Gualdoni et al. recently compared the impact of metformin and AICAR (5-aminoimidazole-4-carboxamide ribonucleoside—a direct pharmacological activator of AMPK) and showed that only AICAR could boost Treg cell differentiation and inhibit Th17 differentiation *in vitro* (175). Combination treatments with metformin have also been trailed in preclinical models of inflammatory disease. For example, Yin et al. reported that CD4^+^ T cells isolated from systemic lupus erythematosus (SLE) patients displayed increased glycolysis and OXPHOS. Simultaneous blockade of these two pathways with 2-deoxyglucose (2DG) and metformin normalized T-cell metabolism and reversed disease biomarkers in a mouse model of SLE (176). In addition, wild-type mice treated with 2DG and metformin did not show signs of toxicity and maintained normal immune function (176).

As previously mentioned, low plasma and tissue glutamine concentrations have been associated with inflammatory conditions, including sepsis and Crohn’s disease. In addition, glutamine has been shown to have beneficial effects on intestinal barrier integrity by enhancing enterocyte proliferation and protecting against enterocyte apoptosis (177). These observations led to the hypothesis that glutamine supplementation may be beneficial for inflammatory conditions, particularly in those disease relating to impaired gut function. However, the clinical benefit of gluta-mine supplementation remains controversial, and a meta-analysis of clinical trials in critically ill patients failed to identify a significant positive effect of glutamine supplementation (178). Moreover, a glutamine-enriched diet in pediatric patients with Crohn’s disease even leads to increased disease activity in some of the subjects (179). Given that the glutamine metabolite α-ketoglutarate was shown to promote Th1 differentiation (101), the potential benefits of glutamine on promoting mucosal barrier health may be negated by the immune-enhancing effects of glutamine on Teff cells.

The therapeutic strategies mentioned earlier illustrate the immunomodulatory potential of targeting metabolic pathways. However, it remains to be explored how targeting T cell metabolism will affect the function of other immune subsets, such as B cells and innate immune cells, which also play a key role in driving chronic inflammatory diseases.

## Concluding Remarks

In chronic inflammatory diseases, T cells infiltrate and are retained at the affected site where they drive the destruction of the surrounding tissues. The inflammatory environment is a site of active leukocyte proliferation; however, here, the cells are exposed to numerous restrictive factors: hypoxic conditions, high lactate, low pH, decreased glucose and amino acids concentrations as well as increased levels of ROS. Nutrient deprivation might actually be a mechanism through which the environment regulates T cell function. The evidence presented in this review promotes the concept that Treg cells seem better equipped than Teff cells to handle nutrient starvation. The active recruitment and proliferation of leukocytes at the inflamed tissue may also contribute to the limited availability of nutrients, allowing the balance to be tilted toward Treg activity that could temper the inflammatory response.

Although we do not have much data on how nutrient availability is altered in inflamed human tissues, we can obtain some useful information by metabolic profiling of biologic samples, such as serum or urine. A recent study performed a large-scale profiling of the urine metabolome from patients with six different common inflammatory diseases: RA, psoriatic arthritis, psoriasis, SLE, Crohn’s disease, and ulcerative colitis (180). They found that decreased concentrations of urine citrate, an intermediate in the TCA cycle, correlated with high disease activity in patients compared with controls (180). Several studies looking at sera samples from RA patients highlighted distinct metabolic features, including decreased levels of amino acids and glucose, along with increased levels of fatty acids and cholesterol (181–183). Moreover, metabolomic studies of the serum of MS patients revealed an excess of ROS and reactive nitrogen species (184, 185), and recent studies reported that increased glucose metabolites in the cerebrospinal fluid and serum of MS patients were positively correlated with disease progression and activity (186, 187).

A key question that arises from the studies presented in this review is how closely do *in vitro* culture models recapitulate the *in vivo* microenvironment conditions present at inflammatory sites? The vast majority of *in vitro* assays are performed at nutrient and oxygen levels that are higher than those observed in tissue. Thus, the metabolic influence that the inflammatory environment exerts on T cell function *in vivo* may account for experimental inconsistencies observed between T cell responses *in vitro* and in animal models. In addition, studies often assess the impact of single factors on T cell function, for example, either hypoxia or glucose depletion. However, these factors should also be analyzed in combination to establish a better understanding of the dynamic and synergistic effects, the inflammatory landscape plays on T cells. As discussed earlier, many metabolic activities are regulated to some extent by circadian rhythms, and several experimental studies have shown that immune parameters, including T cell responses, can vary based on the time of day procedures are performed (73, 188, 189). These observations suggest that such metabolic fluctuations could impact on the reproducibility of immunological data across experiments, and this should be considered during experimental design, particularly for *in vivo* experiments in rodents.

**Figure 3:**
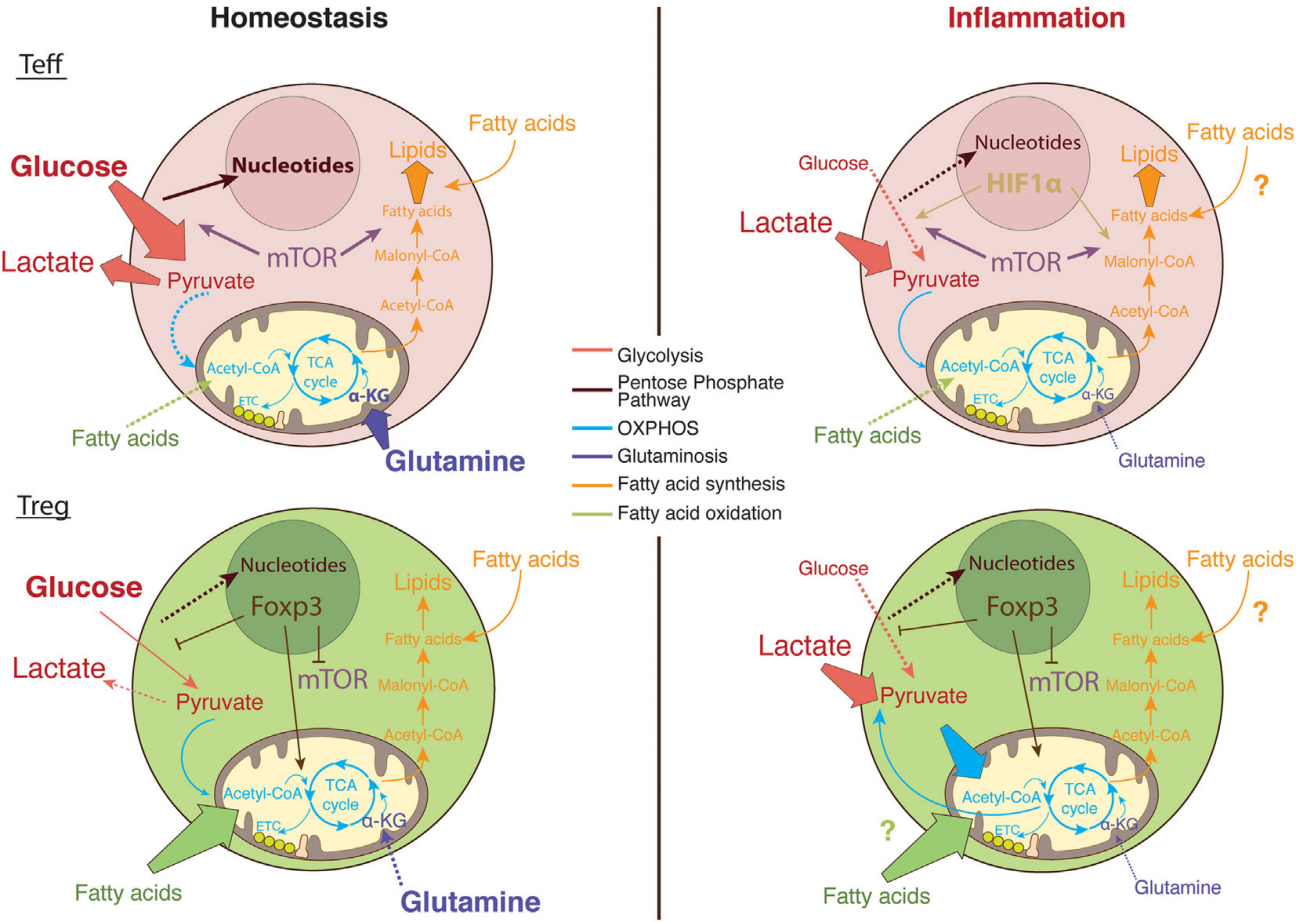
The metabolic pathways utilized by T cells in nutrient replete versus metabolically challenging environments. Under homeostatic conditions, glucose enters the cytosol of activated T cells and is converted into pyruvate *via* glycolysis (89). Pyruvate can either be further metabolized into lactate and secreted or entered into the tricarboxylic acid (TCA) cycle to generate ATP *via* the electron transport chain (ETC) in the process of oxidative phosphorylation (OXPHOS) (120). Moreover, the glucose breakdown intermediates produced during glycolysis can be metabolized *via* the pentose phosphate pathway donating important building blocks for nucleotide and amino acid synthesis (89). Activated Teff cells rapidly augment fatty acid synthesis while concomitantly decreasing fatty acid oxidation (FAO). In comparison, regulatory T (Treg) cells actively take up exogenous fatty acids to sustain their proliferation through FAO (111). In addition, Teff cells rely on glycolysis and glutaminolysis to proliferate and obtain the necessary cofactors for survival while Tregs differentiation is enhanced in glutamine-deprived environments (3, 101). Foxp3 plays a major role in regulating the metabolic pathways necessary for Treg cells function by upregulating OXPHOS activity (149, 150), blocking mechanistic target of rapamycin (mTOR) signaling (156), and glycolysis (149). In the inflamed tissue, in low oxygen conditions, Teff cells upregulate survival mechanisms including the oxygen-sensitive transcription factor, hypoxia-inducible factor (HIF)-1α. HIF-1α promotes anaerobic metabolism through increased expression of glucose transporters, as well as induction of glycolytic enzymes (4, 45). However, glucose supply is limited in the inflamed tissue while lactate is abundant; therefore, T cells favor the conversation of lactate to pyruvate. Tregs are able to sustain the use of lactate-derived pyruvate as a source of energy through the generation of NAD^+^ during OXPHOS (149).

It remains to be fully determined how the complex T cell metabolic machinery handles the microenvironment at the inflamed site and how this shapes T cell intracellular signaling pathways and gene transcription (Figure 3). Understanding how the metabolic pathways that fuel T cell function and proliferation differ within the inflammatory environment may lead to targeted therapeutic strategies for chronic inflammatory diseases, with a few of these therapies already shown promising preliminary results.

## Author Contributions

CD, AK, and KM all contributed to the writing of this review.

## Conflict of Interest Statement

The authors declare that the research was conducted in the absence of any commercial or financial relationships that could be construed as a potential conflict of interest.
